# The thyroid function of Graves' disease patients is aggravated by depressive personality during antithyroid drug treatment

**DOI:** 10.1186/1751-0759-5-9

**Published:** 2011-08-09

**Authors:** Atsushi Fukao, Junta Takamatsu, Sumihisa Kubota, Akira Miyauchi, Toshiaki Hanafusa

**Affiliations:** 1 Ibaraki City Public Health Medical Center, 3-13-5 Kasuga, Ibaraki, Osaka, Japan; 2 Takamatsu Thyroid Clinic, Takatsuki, 7-27-101 Konyacho, Takatsuki, Osaka, Japan; 3 Kuma Hospital, 8-2-35, Shimoyamate-dori, Chuoku, Kobe, Hyogo, Japan; 4 Department of Internal Medicine (I), Osaka Medical College, 2-7 Daigakumachi, Takatsuki, Osaka, Japan

## Abstract

**Background:**

We previously reported that depressive personality (the scores of hypochondriasis, depression and psychasthenia determined by the Minnesota Multiphasic Personality Inventory (MMPI)) and daily hassles of Graves' disease (GD) patients treated long trem with antithyroid drug (ATD) were significantly higher in a relapsed group than in a remitted group, even in the euthyroid state. The present study aims to examine the relationship among depressive personality, emotional stresses, thyroid function and the prognosis of hyperthyroidism in newly diagnosed GD patients.

**Methods:**

Sixty-four untreated GD patients responded to the MMPI for personality traits, the Natsume's Stress Inventory for major life events, and the Hayashi's Daily Life Stress Inventory for daily life stresses before and during ATD treatment.

**Results:**

In the untreated thyrotoxic state, depressive personality (T-scores of hypochondriasis, depression or psychasthenia greater than 60 points in MMPI) were found for 44 patients (69%). For 15 (23%) of these patients, the scores decreased to the normal range after treatment. However, depressive personality persisted after treatment in the remaining 29 patients (46%). Normal scores before treatment were found for 20 patients (31%), and the scores were persistently normal for 15 patients (23%). The remaining 5 patients (8%) had higher depressive personality after treatment. Such depressive personality was not associated with the severity of hyperthyroidism. Serum TSH receptor antibody activity at three years after treatment was significantly (p = 0.0351) greater in the depression group than in the non- depression group. The remission rate at four years after treatment was significantly (p = 0.0305) lower in the depression group than in the non- depression group (22% vs 52%).

**Conclusion:**

The data indicate that in GD patients treated with ATD, depressive personality during treatment reflects the effect of emotional stress more than that of thyrotoxicosis and that it aggravates hyperthyroidism. Psychosomatic therapeutic approaches including antipsychiatric drugs and/or psychotherapy appears to be useful for improving the prognosis of hyperthyroidism.

## Background

There are many reports that emotional stress is related to the onset of hyperthyroidism due to Graves'disease (GD)[1-4]. Some reports suggest that emotional stress is also related to the exacerbation and relapse of hyperthyroidism [5-8]. On the other hand, there are many reports about the existence of various mental disorders such as irritability, anxiety, depression and mania in GD patients [9,10]. Generally speaking, such disorders are thought to be caused by the thyrotoxic condition and could be improved after treatment because thyroid hormones have chemical effects to psychological states including reinforcement of β-adrenergic effects. In fact, in small studies, only an antithyroid drug (ATD) or β blocker improved depression and anxiety associated with GD [11,12]. However, in bigger studies, prevalence of anxiety, depression and cognitive disturbance were often seen in patients in a euthyroid state after treatment [13-15].

Previously, we have reported that depressive personality (the scores of hypochondriasis, depression and psychasthenia determined by the Minnesota Multiphasic Personality Inventory (MMPI) [16]) and daily hassles of GD patients who underwent long-term ATD treatment were significantly higher in a relapsed group than in a remitted group even in the euthyroid state [8]. These data suggest that depressive personality in GD during treatment is caused by not only thyrotoxicosis but also other factors including premorbid disease personality and emotional stresses. Alternatively, our data suggest that depressive personality during treatment aggravates the prognosis of ATD treated hyperthyroidism. To clarify the relationship among depressive personality, thyroid function, emotional stresses and the prognosis of hyperthyroidism, we have done this prospective study to reanalyze the relationships in new, untreated GD patients.

## Methods

Eighty newly diagnosed GD patients were asked to fill out questionnaires at two timings; when in a thyrotoxic state before ATD treatment and in a euthyroid state during ATD treatment (after 6-12 months: average 7 months). The questionnaires; contained several measures of psychological factors and were mailed back to the authors. Of the patients contacted, all 80 signed a consent form, and 64 (10 males and 54 females, 34.5 ± 13.2 years old) responded. The MMPI, the Natsume's Stress Inventory [17] and the Hayashi's Daily Stress Inventory [18,19] were given to all participants. Depressive personality was diagnosed when the T-scores of hypochondriasis, depression or psychasthenia were greater than 60 points on the MMPI, according to the results of our former study. The relationships among depressive personality, thyroid function, and emotional stresses (life events and daily life stress) were then evaluated. The relationships between depressive personality and the prognosis of hyperthyroidism was also evaluated prospectively in 48 patients who could be followed for 48 months, excluding 16 patients who dropped out. The initial dose of ATD was thiamazole 15~30 mg/day according to the level of thyrotoxicosis of each patient, and the dose of ATD was gradually redused to maintain a euthyroid state. Thiamazole was changed to propylthiouracil due to a severe drug reaction by one patient. When, after ATD treatment for at least 12 months, normal serum free T4 and TSH concentrations continued for more than 6 months with thiamazole 2.5 mg/day or propylthiouracil 25 mg/day therapy and TRAb activity was negative, ATD treatment was stopped. Patients who maintained normal serum free T4 and TSH concentrations for more than 12 months after ATD withdrawal, excluding relapsed patients, were judged as being inremission.

### Tests for Psychological Assessment

Three questionnaires were presented to each subject. In order to examine ten clinical scales of personality traits, the Japanese version of MMPI with 383 items developed by Tanaka et al [20], originally developed by Hathaway and McKinley was used. The three standard validating scales included lie, validity, correction and the 10 clinical scales including hypochondriasis, depression, conversion hysteria, psychopathic deviation, masculinity and feminity, paranoia, psychasthenia, schizophrenia, hypomania and social introversion.

In order to assess major life events, the Natsume's Stress Inventory developed by Natsume et al with 67 items including 65 events together with two items, "stress at present"and "ability to tolerate stress", was used. The events listed were life events in the following categories: individual life, family life, occupational life and social life. Although their frequency of occurrence was low, their impacts on life and the resultant adaptation were usually considerable. Examples include "death of spouse", "pregnancy", "retirement" or "company failure". In this inventory, 42 events were similar to the "schedule of recent events" created by Holmes and Rahe [21], and 23 events were newly added to suit Japanese culture. The studied subjects answered major life events in the previous 12 months and rated the impact of each event as 0 to 100 points for comparison with a score of 50 points for the reference-standard of stress strength of "marriage".

In order to assess daily hassles (annoyances) and daily uplifts (positive experiences), the Hayashi's Daily Life Stress Inventory with 71 items developed by Hayashi et al, was used. This inventory was modified from Lazarus et al's [22] original questionnaire scales for "daily hassles" and "daily uplifts" to suit the Japanese life style. Daily hassles included, for example, the 8 valid scales including the following "annoyed about life with others", "annoyed by lack of time" or "unfulfilled in personal life". Daily uplifts included "satisfaction with work", "enough time available", or "peaceful in home life". Subjects answered daily minor events in the last 12 months and rated the impact of each event as 1 to 3 points. Two scores were generated; first, the total number of daily minor events encountered, and second, the hassles scores and uplifts scores by intensities, i.e. summing the scales per degree rated.

### Thyroid related tests

Serum free T4 and TSH concentrations were determined by enzyme immunoassay (Dainabott Co.). Serum free T3 concentrations was determined by radio immunoassay (Ortho Clinical Diagnosis Co.). Serum TRAb activity was determined by radioreceptorassay (Cosmic Co.). Thyroid volume was determined by ultrasonography according to our reported method [23] [Thyroid volume (ml) = 0.7 × (r-width(mm) × r-thickness(mm) × r-length(mm) + l-width(mm) × l-thickness(mm) × l-length(mm))]. The diameter of the thyroid gland was determined by calipers. Serum free T4 and TSH concentrations were examined every 1 to 3 months during ATD treatment and the amount of ATD was regulated to normalize serum free T4 concentration. Serum TRAb activity and the diameter of the thyroid gland were examined before treatment, and, 6,12, 24, 36, and 48 months during treatment.

### Statistical Analysis

Grouped data were expressed by mean ± SD. All data were compared by Student t-test, Mann-Whitney's test or chi-square test for independence, expressed by p value. When there were no significant differences of variance analyzed by Barlett test, the data were also compared by one factor analysis of variance (ANOVA).

## Results

### 1. The changes of depressive personality between before and during treatment (Figure 1)

**Figure 1:**
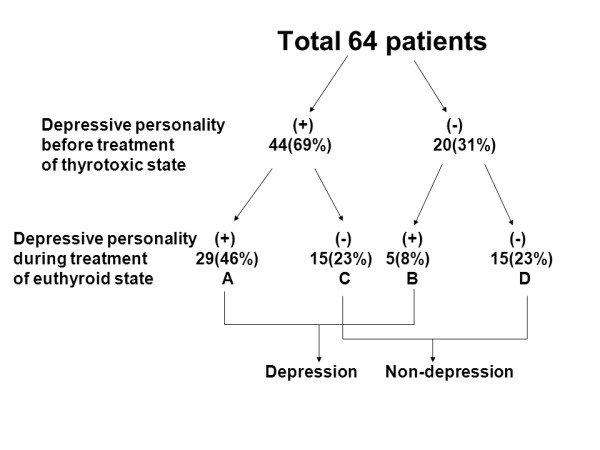
**Changes of the depressive personality of Graves' disease patients before and during treatment**. GroupA: depressive personality was present before and persisted after treatment. GroupB: depressive personality scores became higher after treatment. GroupC: depressive personality was present before treatment and decreased to within the normal range after treatment. GroupD: depressive personality did not appear either before or after treatment.

In the untreated thyrotoxic state, depressive personality was found in 44 patients (69%). Of them, 15 (23%) had scores decreased to normal range after treatment (groupC). However, in the remaining 29 patients (46%), the depressive personality persisted after treatment (groupA). Normal scores in the untreated state were found for 20 patients (31%), and the scores were continuously normal for 15 patients (23%) (groupD), while the remaining 5 patients (8%) had higher depressive personality scores after treatment (groupB).

### 2. The relationships among depressive personality, thyroid function, and emotional stresses

**Figure 2:**
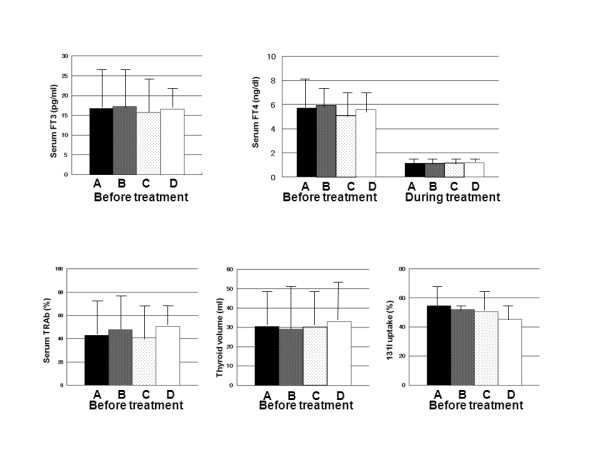
**Comparisons of thyroid functions and severity of hyperthyroidism among four groups**. The data from each group are shown as mean ± SD. GroupA(29): depressive personality was present before and persisted after treatment. GroupB(5): depressive personality scores became higher after treatment. GroupC(15): depressive personality was present before treatment and decreased to within the normal range after treatment. GroupD(15): depressive personality did not appear either before or after treatment. There were no significant differences in any parameters among the four groups by ANOVA.

**Figure 3:**
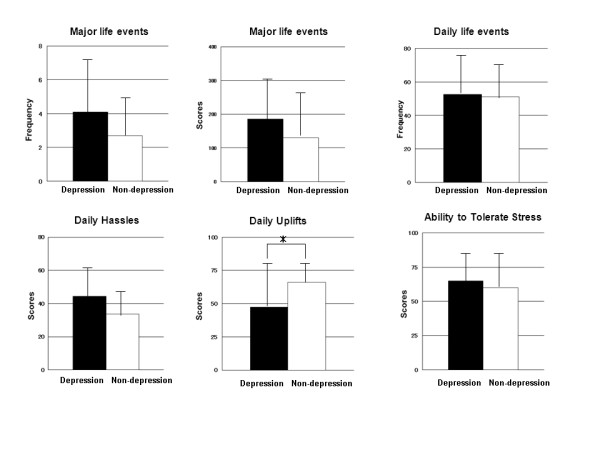
**Comparisons of emotional stresses between the depression and non-depression groups**. The closed bar express the depression group (34) and open bar express the non- depression group (30). The data from each group are shown as mean ± SD. Significant difference: *P < 0.005 by Mann-Whitney's test.

Among the four groups of patients (groups A~D), there were no differences in pre and post treatment serum FT4 concentrations, or in pre treatment serum FT3 concentrations, serum TRAb activity, thyroid volume or ^131^I-uptake (Figure 2). Thirty four patients with depressive personality, even in the euthyroid state (group A and B) had significantly (p = 0.003) lower daily uplifts than the remaining 30 patients without depressive personality (group C and D) (Figure 3).

### 3. Comparison of the prognosis of hyperthyroidism between the depression and non- depression groups. (Figure 4)

**Figure 4:**
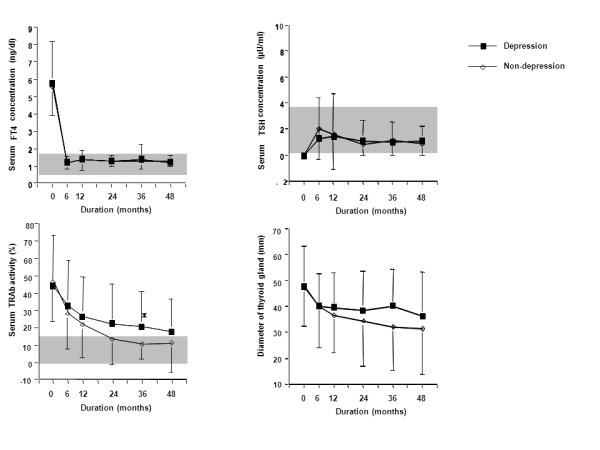
**Comparison of the prognosis of hyperthyroidism between the depression and non-depression groups**. The data from each group are shown as mean ± SD. The gray zone express the normal ranges. Significant difference: *P < 0.05 by Student t-test. Remission rate: depressive group 22% (5/23) vs non-depressive group 52% (13/25) (P < 0.05 by chi-square test).

Forty-eight patients were followed for four years. There were no differences in serum FT4 and TSH concentrations before or during treatment between the two groups. Serum TRAb activity at three years after ATD treatment was significantly (p = 0.0351) greater in the 23 patients with depressive personality than in the 25 patients without depressive personality (Figure 4). The rate of remission at four years after treatment was significantly (p = 0.0305) lower in the depression group than in non- depression group (22% vs 52%).

## Discussion

The principal finding of our study was that there were many GD patients who had depressive personality even when euthyroid during treatment. If such mental abnormalities during treatment were only sequelae of thyrotoxicosis, it would be expected that these abnormalities would have a tendency to improve after treatment and a tendency to continue more often in patients with greater thyrotoxicosis. However, in our study, depressive personality before treatment improved in only 15 of 44 patients and it appeared for the first time after treatment in 5 patients. Additionally, whether or not depressive personality exists is not associated with either thyroid function or severity of hyperthyroidism, including TRAb activity, thyroid volume and ^131^I uptake. On the other hand, the patient group with depressive personality had a lower prevalence of daily uplifts than did the patient group without depressive personality. These results suggest that such depressive personality as seen in GD is caused by emotional stresses or premorbid personality rather than by the chemical effects of the thyroid hormone, contrary to former consensus.

Secondly, the rate of remission at four years after ATD treatment was significantly lower in the depression group than in the non- depression group. These results suggest that depressive personality, even when euthyroid during treatment, in GD is related to the aggravation of hyperthyroidism. Because depressive patients experience more daily hassles and fewer daily uplifts than non-depressive patients, emotional stresses may aggravate hyperthyroidism by the neuro- immuno-endocrine system [24] in depressive patients. Although in this study remission was judged by normal serum free T4 and TSH concentrations for more than 12 months after ATD withdrawal, a time interval of 12 months may be too short to prove stable remission. Further observation should be made. Controls including healthy population groups or patiens with nonautoimmune hyperthyroidism will be necessary in future studies to clarify the relationships.

Recently, we have experienced three cases of first remission after long term ATD treatment together with antidepressants in GD patients with depression [25]. There are brief reports in which the administration of a minor tranquilizer together with ATD increased the remission rate of hyperthyroidism [26,27]. In another study [28], we found that rational thinking and expressing feelings are associated with early remission in ATD treated GD patients. Psychotherapy for improving rational thinking and expression of feelings may also be useful in improving the prognosis in GD patients with depression [29].

In conclusion, our findings suggest that in ATD treated GD patients, depressive personality during treatment when patients are euthyroid reflects the effect of emotional stresses rather than thyrotoxicosis and that it aggravates hyperthyroidism. Intervention studies with psychosomatic therapeutic approaches, including antipsychiatric drugs and psychotherapy, should be carried out with large numbers of patients.

## Competing interests

The authors declare that they have no competing interests.

## Authors' contributions

AF conceived the study, participated in the design of the study, carried out data collection, performed the statistical analysis and drafted the manuscript. JT participated in the design of the study and reviewed the manuscript. SK participated in the coordination of the study and reviewed the manuscript. AM participated in the coordination of the study and reviewed the manuscript. TH participated in the coordination of the study and reviewed the manuscript. All authors read and approved the final manuscript.
